# Plasma Lipidomics Profiling to Identify the Biomarkers of Diagnosis and Radiotherapy Response for Advanced Non-Small-Cell Lung Cancer Patients

**DOI:** 10.1155/2024/6730504

**Published:** 2024-01-27

**Authors:** Minghe Lv, Shali Shao, Yajing Du, Xibing Zhuang, Xiangdong Wang, Tiankui Qiao

**Affiliations:** ^1^Center for Tumor Diagnosis and Therapy, Jinshan Hospital, Fudan University, Jinshan District, Shanghai 201508, China; ^2^Department of Radiotherapy, Shuguang Hospital Affiliated to Shanghai University of Chinese Traditional Medicine, Pudong New Area, Shanghai 201203, China

## Abstract

**Background:**

Advanced lung cancer that contributes to a heavy burden on medical institutions is the leading cause of cancer-related death and is often accompanied by metabolic disorders. In this study, we aimed to explore the biomarkers of diagnosis and radiotherapy response in non-small-cell lung cancer (NSCLC) patients by plasma lipidomics analysis.

**Method:**

Using triple-quadrupole mass spectrometer analysis, our research characterized the plasma lipid metabolomics profile of 25 healthy controls and 31 advanced NSCLC patients in each of three different radiotherapy phases.

**Results:**

The results showed altered lipid elements and concentrations among NSCLC patients with different radiotherapy phases, NSCLC subtypes, and different radiotherapeutic responses. We found that compared to the healthy controls, myelin-associated glycoprotein (MAG), phosphatidylinositol (PI), and phosphatidylserine (PS) were mainly and significantly altered lipid elements (> twofold, and *p* < 0.05) among NSCLC patients with different radiotherapy phases. Through comparison of lipid elements between bad and good responses of NSCLC patients with radiotherapy, the obviously declined phosphatidylglycerol (PG 18 : 0/14 : 0, 18 : 1/18 : 3, and 18 : 0/20 : 1) or markedly elevated PI (20 : 0/22 : 5 and 18 : 2/22 : 4) and phosphatidic acid (PA 14 : 0/20 : 4, 14 : 0/20 : 3, and 18 : 2/22 : 4) could indicate poor therapeutic response for NSCLC patients. The results of ROC curve analysis suggested that PG (18 : 0/20 : 1 and 18 : 0/14 : 0) could clearly predict the radiotherapeutic response for NSCLC patients, and PS (18 : 0/20 : 0) and cholesterol were the first two lipid components with the most potential for the diagnosis of advanced NSCLC.

**Conclusion:**

Our results indicated that plasma lipidomics profiling might have a vital value to uncover the heterogeneity of lipid metabolism in healthy people and advanced NSCLC patients with different radiotherapy phase, and further to screen out radiotherapeutic response-specific biomarkers.

## 1. Background

Lung cancer is the leading cause of cancer-associated death worldwide, and its incidence also ranks among the best [1]. Non-small-cell lung cancer (NSCLC) accounts for approximately 85% of all lung cancer cases [2, 3], and most patients are locally advanced stages by the time they are diagnosed. The standard of care for locally advanced NSCLC is concurrent chemoradiation therapy, which produces a median survival time of 28.7 months [4]. Many studies have shown that radiation treatment can affect changes in metabolism in the body of cancer patients, including head and neck cancer [5] and lung cancer [6, 7]. Abnormal metabolism is one of the critical characteristics of tumor in cell experiments, whereas metabolic variations in radiotherapy-related patients remain elusive. Therefore, identifying the metabolic changes of NSCLC patients in radiation therapy and their relationship are critical, which deserve further investigation.

Metabolomics in cancer provide important information on pathophysiology beyond genomics and proteomics data [8], of which a subset is lipidomics defined as a study about the content and function of whole lipids in the cell or tissue in biological systems. Lipids mainly consist of subclasses of phosphatidic acid (PA), phosphatidylcholines (PC), phosphatidylethanolamine (PE), phosphatidylglycerol (PG), phosphatidylinositol (PI), phosphatidylserine (PS), cholesterol, myelin-associated glycoprotein (MAG), glycerol-based phospholipids, and ceramide-based sphingolipids [9], which have multiple vital biological functions [10], such as biomembrane composition, survival, proliferation, migration, apoptosis, signal transduction, and posttranslational modification [11, 12]. Dysfunction of lipid metabolism was found to be associated with pathogenesis of many diseases, including cancer [13]. Several previous research groups have investigated lipid alterations in early lung cancer patients to elucidate the disease and discover potential biomarkers [14–16]; however, the value of lipid alterations for diagnosis and radiotherapeutic response biomarkers in advanced NSCLC patients is still unclear until now. Therefore, it is significant to investigate the characteristic alterations of lipidomics, explore the biomarkers of diagnosis and radiotherapeutic response, and elucidate possible molecular mechanism in NSCLC patients.

In this article, we aimed to elucidate the values of lipidomics network layers and further to explore the biomarkers of diagnosis and radiotherapeutic response in NSCLC patients. In addition, we attempted to expound the underlying mechanism that ionizing irradiation affected the lipid metabolism in advanced NSCLC patients.

## 2. Methods and Materials

### 2.1. Healthy Controls and NSCLC Patients

From June 2020 to June 2021 at Jinshan Hospital affiliated to Fudan University, peripheral blood was obtained from 31 NSCLC patients (24 males and 7 females; median age was 66 (38-83) years; 20 adenocarcinoma and 11 squamous carcinoma) before radiotherapy (preradiotherapy, pre-RT), during radiotherapy (RT), and after radiotherapy (postradiotherapy, post-RT), and 25 healthy controls (4 males and 21 females; median age was 38 (22-62) years). According to the eighth edition of the TNM Classification of Lung Cancer [17], all NSCLC patients were diagnosed with locally advanced (Stage III, 7) or metastatic (Stage IV, 24) disease. The therapeutic response of target lesions was evaluated by RECIST criteria [18]. We defined CR (complete response) and PR (partial response) as the good response group and defined SD (stable disease) and PD (progressive disease) as the bad response group (16 patients belong to the good response group, and 15 patients belong to the bad response group). All patients and healthy controls were provided a written informed consent, and the study was approved by the ethics committee of Jinshan Hospital affiliated to Fudan University, and the ethical code is IEC-2020-S01.

### 2.2. Isolation of Peripheral Blood Plasma

We collected the fresh peripheral blood of healthy volunteers and NSCLC patients by an EDTA-anticoagulated tube. The blood samples were placed in a centrifuge and centrifuged at 1500 rpm for 5 minutes to obtain the supernatant, namely, plasma, which was then packed in a centrifuge tube and stored at -80°C.

### 2.3. Extract, Purification, and Identification of Lipids from Plasma for Mass Spectrometry Analysis

Approximately 20 *μ*L of thawed plasma was collected into a centrifuge tube. To this, 11 *μ*L of internal standard (Avanti Lipids Polar (Alabaster, AL, USA)) and 700 *μ*L of isopropyl alcohol (Merck Millipore, Billerica, MA, USA) were added. The mixture in the centrifuge tube was then placed on an oscillator and shaken for 1 minute to ensure complete mixing. Afterward, the mixture was refrigerated at -20°C overnight. The next day, the centrifuge tube was subjected to centrifugation at 14000 rpm for 20 minutes. The resulting supernatant was carefully collected, while the precipitate was discarded. The obtained supernatant sample was subjected to a second centrifugation step at 14000 rpm for 20 minutes. Subsequently, the supernatant was transferred to a glass bottle for further analysis. The supernatant sample was tested using a normal-phase liquid chromatography coupled triple-quadrupole mass spectrometer (QTRAP 6500, SCIEX, Framingham, MA, USA) with positive and negative electrospray ionization mode. For lipid extraction, Ultimate SiO2 (250 mm × 4.6 mm, 5 *μ*m; Agilent Technologies, Santa Clara, CA, USA) was utilized, with a flow rate of 1.5 mL/min and high-purity helium. Additionally, 2.0 *μ*L of the sample was added with a split ratio of 50 : 1 at an ignition chamber temperature of 220°C and an injection port temperature of 150°C. The temperature was initially set at 150°C and gradually increased (4°C/min) to 250°C, maintaining this temperature for 5 minutes. The mass spectrometry analysis was conducted using a liquid chromatography-mass spectrometer (FOCUS DSQTM II, Thermo Fisher Scientific) under the following conditions: electron ionization (EI) as the ionization source, ion source temperature at 200°C, ionization voltage at 70 eV, multiplier voltage at 0.9 kV, a 4-minute solvent delay, and a scan range of 50-650 amu. The lipid extract was loaded onto an Ultremex silica gel column (250 mm × 4.6 mm, 5 *μ*m; Phenomenex, Torrance, CA, USA) and eluted using an elution gradient of 300 nL. The gradient started with phase B at 50% from 0 to 5 minutes, then increased to 100% from 5 to 30 minutes, linearly ramped for 10 minutes, and finally returned to 50% from 40 to 41 minutes until completion. The Q-Trap operated in a multiple reaction monitoring mode, employing different precursor/product ion pairs. This allowed for obtaining possible chemical structures, scanning pairs, and quantitative results of 409 lipids from plasma samples. The peak area of each pair was quantified using multiple reaction monitoring data through the MultiQuant software (AB SCIEX).

### 2.4. Comprehensive Analysis of Lipidomics Data

MetaboAnalyst 5.0 (https://www.metaboanalyst.ca/) was used for multivariate statistical analysis and cluster analysis. According to the 50% missing data criteria, no lipids were filtered out. Prior to analysis, missing values were replaced by the median, and all metabolomic MS intensity data were normalized using the Pareto scale. MetaboAnalyst software was used to conduct volcano plot, bubble diagram, heat map, dimensional reduction, or other five major indicators for the NSCLC patients and healthy people.

### 2.5. Statistical Analysis

The data were expressed as mean ± SE. The mean values of each group were calculated and compared. The statistical significance of the differences between groups was tested by one-way ANOVA, and the differences between two groups were tested by one-way two-tailed *T* test. *p* < 0.05 was considered statistically significant. The value and accuracy of clinical phenotypic specific lipid elements in predicting the efficacy of NSCLC patients after radiotherapy were evaluated by using MetaboAnalyst software to construct biomarker analysis and the subject operating characteristic curve (ROC). The KEGG database was used for enrichment analysis.

## 3. Results

### 3.1. Lipidomics Profiles of NSCLC Patients during Different Stages of Radiotherapy

**Figure 1:**
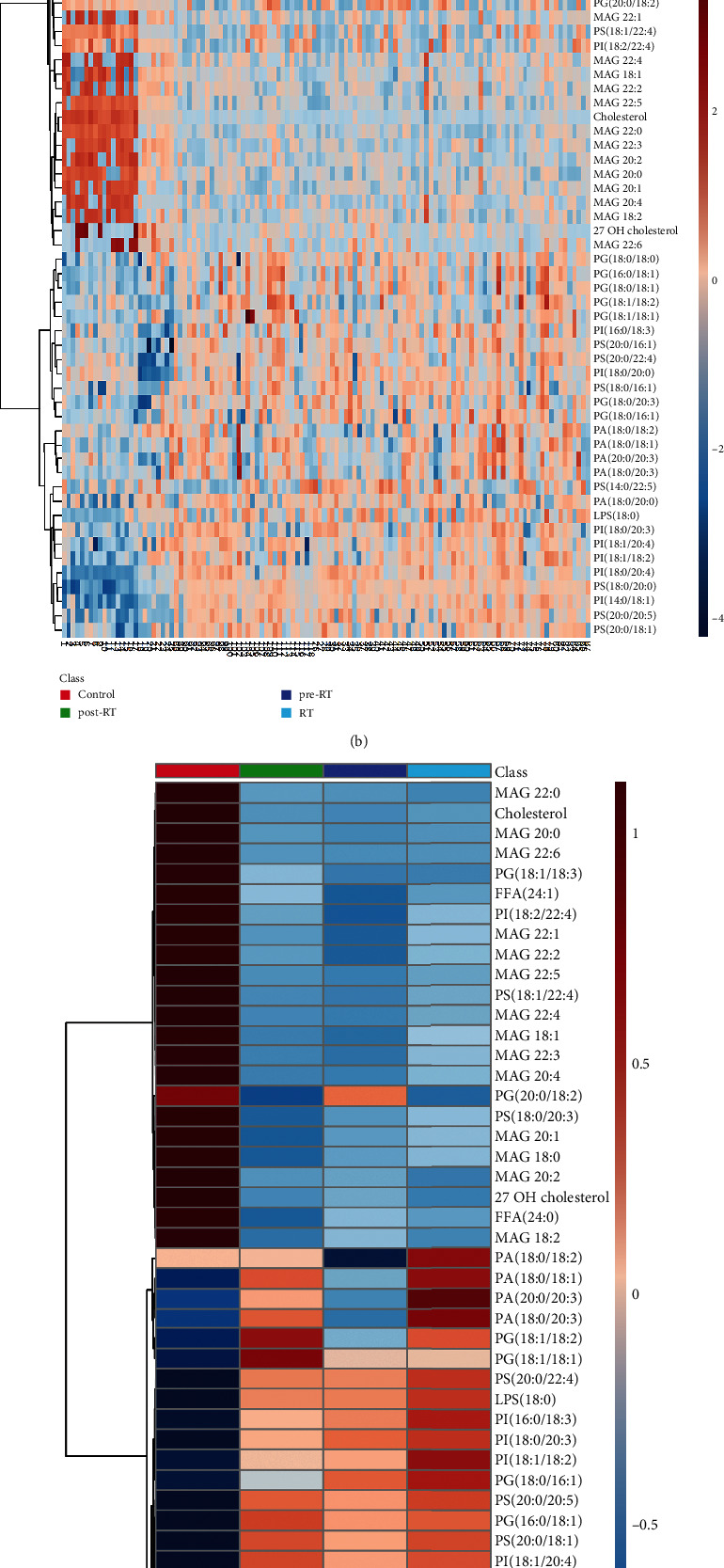
The plot of variable import in project (VIP) and the heat map of the top 50 lipid elements in healthy controls and NSCLC patients in pre-RT, RT, and post-RT groups. (a) The top 15 VIP scores of lipid elements in healthy controls and NSCLC patients were shown by bubble diagram. (b) The top 50 concentration lipid elements in healthy controls and NSCLC patients were exhibited by heat map. (c) The average concentration of lipid elements (top 50) in each group was exhibited by a heat map.

**Figure 2:**
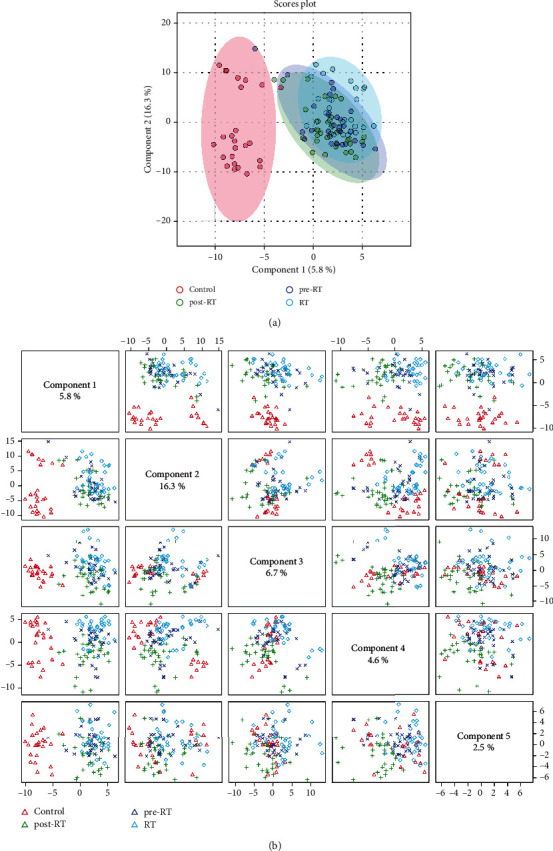
Principal component analysis of lipids in healthy controls and NSCLC patients was performed by partial least squares discrimination analysis (PLS-DA) analysis. (a) Principal component analysis of lipid species and scatters. (b) The histography of five component distributions and percentages.

**Figure 3:**
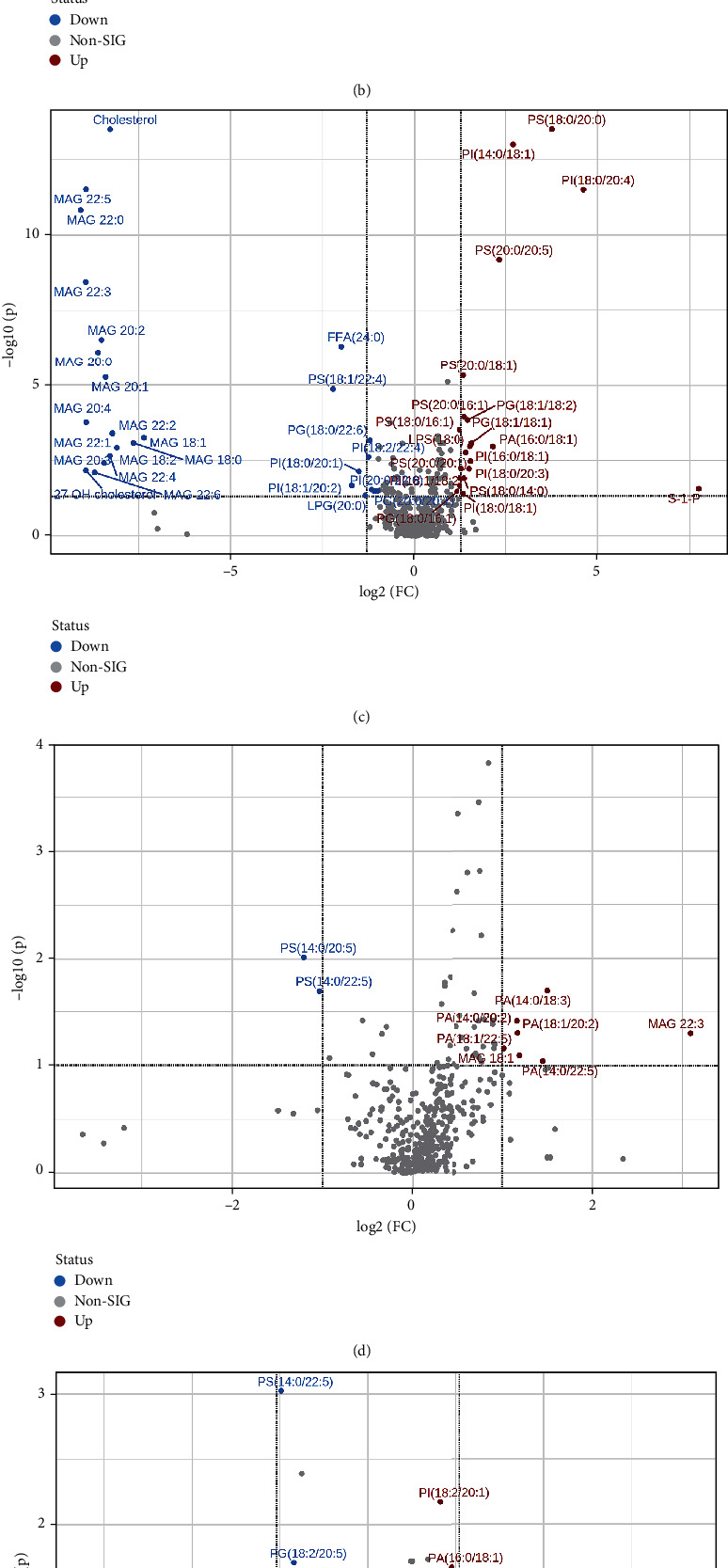
The volcano plots of healthy controls and NSCLC patients during different stages of radiotherapy. (a) The volcano plot between healthy controls and NSCLC patients in pre-RT. (b) The volcano plot between healthy controls and NSCLC patients in RT. (c) The volcano plot between healthy controls and NSCLC patients in post-RT. (d) The volcano plot of NSCLC patients in pre-RT and RT. (e) The volcano plot of NSCLC patients in pre-RT and post-RT. (f) The volcano plot of NSCLC patients in RT and post-RT.

A total of 409 lipid elements of plasma were identified qualitatively and quantitatively, mainly including 20 (4.9%) FFAs (free fatty acids), 13 (3.2%) LPAs (lysophosphosphatidic acids), 16 (3.9%) LPGs (lysophosphatidylglycerols), 16 (3.9%) LPIs (lysophosphatidylinositols), 16 (3.9%) LPSs (lysophosphatidylserines), 77 (18.8%) PAs (phosphosphatidic acid), 77 (18.8%) PGs (phosphatidylglycerols), 76 (18.6%) PIs (phosphatidylinositols), 77 (18.8%) PSs (phosphatidylserines), 2 (0.5%) cholesterols, 17 (4.2%) MAGs (myelin-associated glycoproteins), and 2 (0.5%) sphingosines. As shown in Tables 1 and 2, we found that compared to the healthy controls (> twofold; *p* < 0.05), there were some more than twofold declined or elevated lipid elements of NSCLC patients in pre-RT, RT, or post-RT groups. Compared with the healthy controls, there were 30 more than twofold declined lipid elements in NSCLC patients before radiotherapy, mainly including 46.7% MAG, 20% PI, 10% PG, and 10% PS. Compared with the healthy controls, there were 24 more than twofold declined lipid elements in NSCLC patients during radiotherapy, mainly including 58.3% MAG, 12.5% PG, and 12.5% PS. Compared with the healthy controls, there were 27 more than twofold declined lipid elements in NSCLC patients during radiotherapy, mainly including 55.6% MAG, 14.8% PI, and 7.4% PG. Compared to healthy controls, the majority of those elevated lipid elements were 43.75% PS and 25% PI in NSCLC patients of pre-RT group; 45% PI, 20% PS, and 15% PG in NSCLC patients of RT group; and 35% PI, 35% PS, and 20% PG in NSCLC patients of post-RT group. From the above, we concluded that compared with normal subjects, the markedly decreased and statistically significant lipid elements in NSCLC patients at different radiotherapy stages were all MAG, and the obviously increased and statistically significant lipid elements in NSCLC patients at pre-RT, RT, or post-RT were mainly PI and PS. Partial least squares discriminant analysis (PLS-DA) was used to determine the top 15 lipid elements in each group according to variable import in project (VIP) scores. As shown in Figure 1(a), the bubble plot showed that PI (14 : 0/18 : 1), MAG (22 : 0), PS (20 : 0/20 : 5, 20 : 0/18 : 1, and 20 : 0/22 : 4) decreased in NSCLC patients of pre-RT group; PI (18 : 0/20 : 4) and MAG (20 : 0) decreased in NSCLC patients of RT group; PS (18 : 0/20 : 0 and 20 : 0/16 : 1), cholesterol, MAG (22 : 5, 20 : 2, and 22 : 3), and PI (16 : 0/18 : 3 and 18 : 1/18 : 2) decreased in NSCLC patients of post-RT group. Moreover, we found that the top 50 of lipid elements have an obvious distribution in NSCLC patients, as compared with healthy controls (Figure 1(b)). The heat map for an average concentration of the top 50 lipid elements indicated that in the figure from the top down, the expressions of the first 20 lipids in NSCLC patients were significantly lower than that in healthy people, while the expressions of the last 30 lipids in NSCLC patients were significantly higher than that in healthy people (Figure 1(c)). As shown in Figure 2(a), two-dimensional principal component analysis showed that there was a significant difference in lipid species distribution between lung cancer patients and healthy controls. The PLS-DA component analysis exhibited that the five principal components were 5.8%, 16.3%, 13.3%, 6.7%, and 4.6% (Figure 2(b)). The volcano figure with the transverse axis of the log2 (FC) and the longitudinal axis of −log 10 (*p* value), where every dot represents one lipid species of healthy controls or NSCLC patients, indicated that the expression levels of lipid elements markedly elevated or declined between healthy controls and NSCLC patients in pre-RT (Figure 3(a)), RT (Figure 3(b)), or post-RT (Figure 3(c)) group. In the volcanic map, lipids that have changed more than twice as much were identified with statistically significant changes, with blue dots representing declining lipids and red dots representing rising lipids, as compared with healthy people.

### 3.2. Different Lipid Omics between NSCLC Patients in Pre-RT, RT, or Post-RT Groups

**Figure 4:**
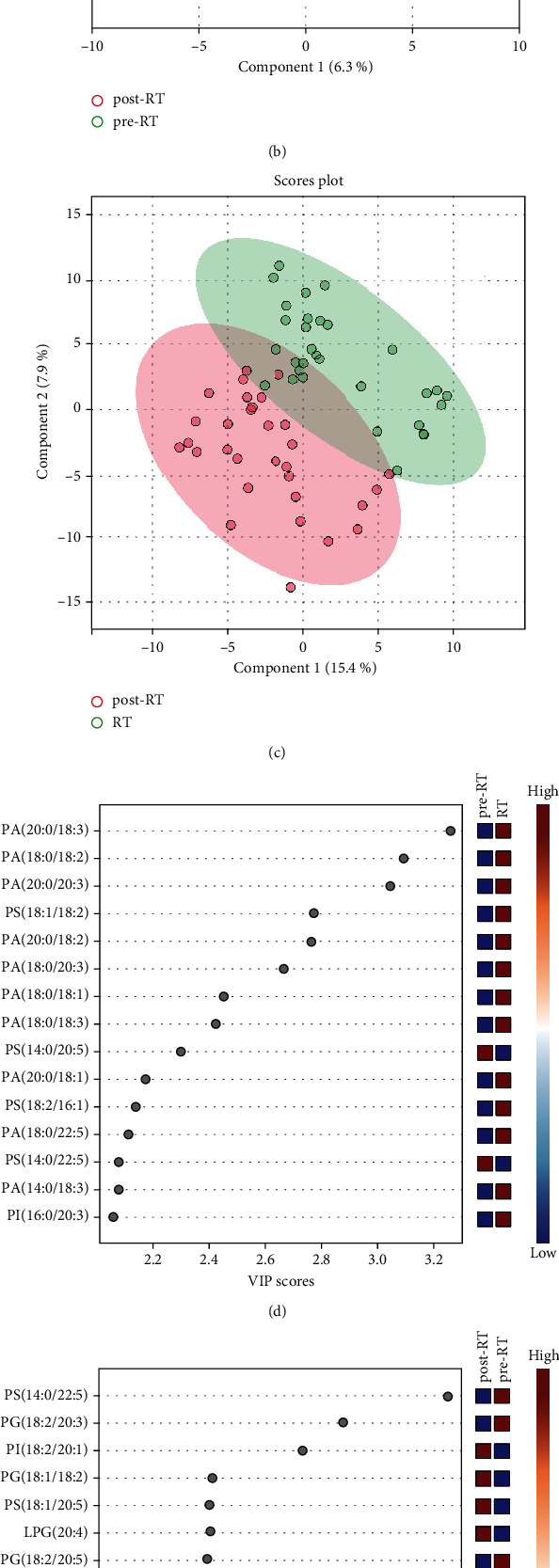
Comparison of lipid elements in NSCLC patients with different stages of radiotherapy. (a–c) The plots of two-dimensional principal component analysis show the lipid species and scatters. (d–f) The bubble diagram shows the VIP scores of the top 15 lipid elements in NSCLC patients with different stages of radiotherapy.

The volcano plots between each group showed that as compared with the pre-RT group, PS (14 : 0/20 : 5 and 14 : 0/22 : 5) or PA (14 : 0/18 : 3, 14 : 0/20 : 2, 18 : 1/20 : 2, 18 : 1/22 : 5, and 14 : 0/22 : 5) and MAG (18 : 1 and 22 : 3) declined or increased more than twofold in the NSCLC patients of RT group (Figure 3(d)); PS (14 : 0/22 : 5) and PG (18 : 2/20 : 5) or PI (18 : 2/20 : 1), PS (14 : 0/20 : 3), and PA (16 : 0/18 : 1) declined or increased more than twofold in the NSCLC patients of post-RT group (Figure 3(e)). PA (18 : 2/22 : 5, 14 : 0/20 : 2, and 18 : 2/18 : 2), MAG (20 : 1), LPS (18 : 1), LPG (20 : 0), and PI (18 : 0/20 : 1) or PG (18 : 1/22 : 6) declined or increased more than twofold in the NSCLC patients of post-RT group, compared to RT group (Figure 3(f)). As compared with NSCLC patients of pre-RT group, about 4 or 2 lipid elements or 3 or 2 lipid elements markedly elevated or decreased more than twofold in NSCLC patients of RT group or post-RT group, respectively. In addition, about 1 or 7 lipid elements markedly elevated or decreased more than twofold in NSCLC patients of post-RT group, as compared with NSCLC patients of RT group, respectively (Table 3). As shown in Figures 4(a)–4(c), the two-dimensional principal component analysis diagram showed significant differences in lipid species distribution between each group, especially between before and after radiotherapy. The bubble diagram about VIP exhibited that compared to NSCLC patients of pre-RT group, 86.7% of the first 15 lipids were elevated in NSCLC patients of RT group and 73.3% of the first 15 lipids were elevated in NSCLC patients of post-RT group; however, as compared with NSCLC patients of RT group, 86.7% of the first 15 lipids are declined in NSCLC patients of post-RT group (Figures 4(d)–4(f)).

### 3.3. Different Lipid Omics between Lung Adenocarcinoma and Lung Squamous Carcinoma

**Figure 5:**
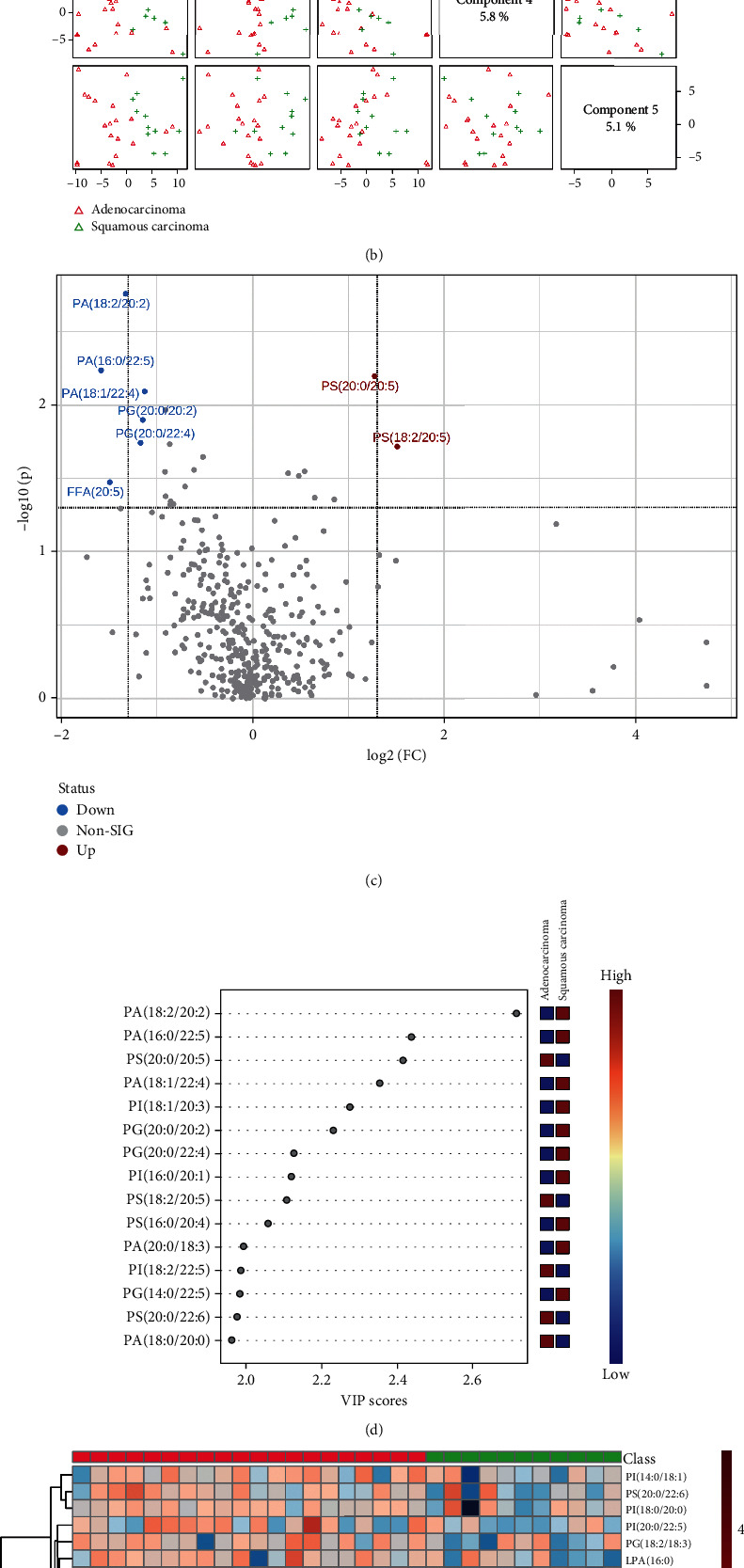
Comparison of alteration of lipid elements between lung adenocarcinoma and lung squamous carcinoma patients before radiotherapy. (a, b) The plots of two-dimensional principal component analysis and five component distributions and percentages show the lipid species and scatters. (c) The volcano shows the changes of lipid elements in the two lung cancer subtypes. (d) The bubble diagram shows the VIP scores of the top 15 lipid elements in the lung adenocarcinoma and lung squamous carcinoma patients. (e) The heat map shows the detailed concentration of lipid elements (top 50).

As compared with lung squamous carcinoma patients of pre-RT, RT, or post-RT groups, about 2 or 7 lipid elements, about 2 or 3 lipid elements, or about 4 or 5 lipid elements markedly elevated or decreased more than twofold in lung adenocarcinoma patients of corresponding group (Table 4). As shown in Figures 5(a) and 5(b), before radiotherapy, lipid types in NSCLC patients with the two pathological types were clearly distinguished on two-dimensional principal component analysis maps, and the PLS-DA component analysis exhibited that the five principal components were 14.9%, 7.3%, 8.2%, 5.8%, and 5.1%.  Figure 5(c) was a visual volcano diagram of lipid elements increased or decreased by more than two times when the two lung cancer subtypes were compared. As shown in Figure 5(d), the bubble plot via PLS-DA component analysis showed the top 15 lipid elements according to the VIP scores from the comparison between lung adenocarcinoma and lung squamous carcinoma of pre-RT group. We found that almost all FFA in the top 50 lipid elements (as shown in Figure 5(e)) did not obviously alter between different lung cancer subtypes, and there were significant differences in the expression of other lipid elements between the two subtypes of lung cancer in pre-RT group.

**Figure 6:**
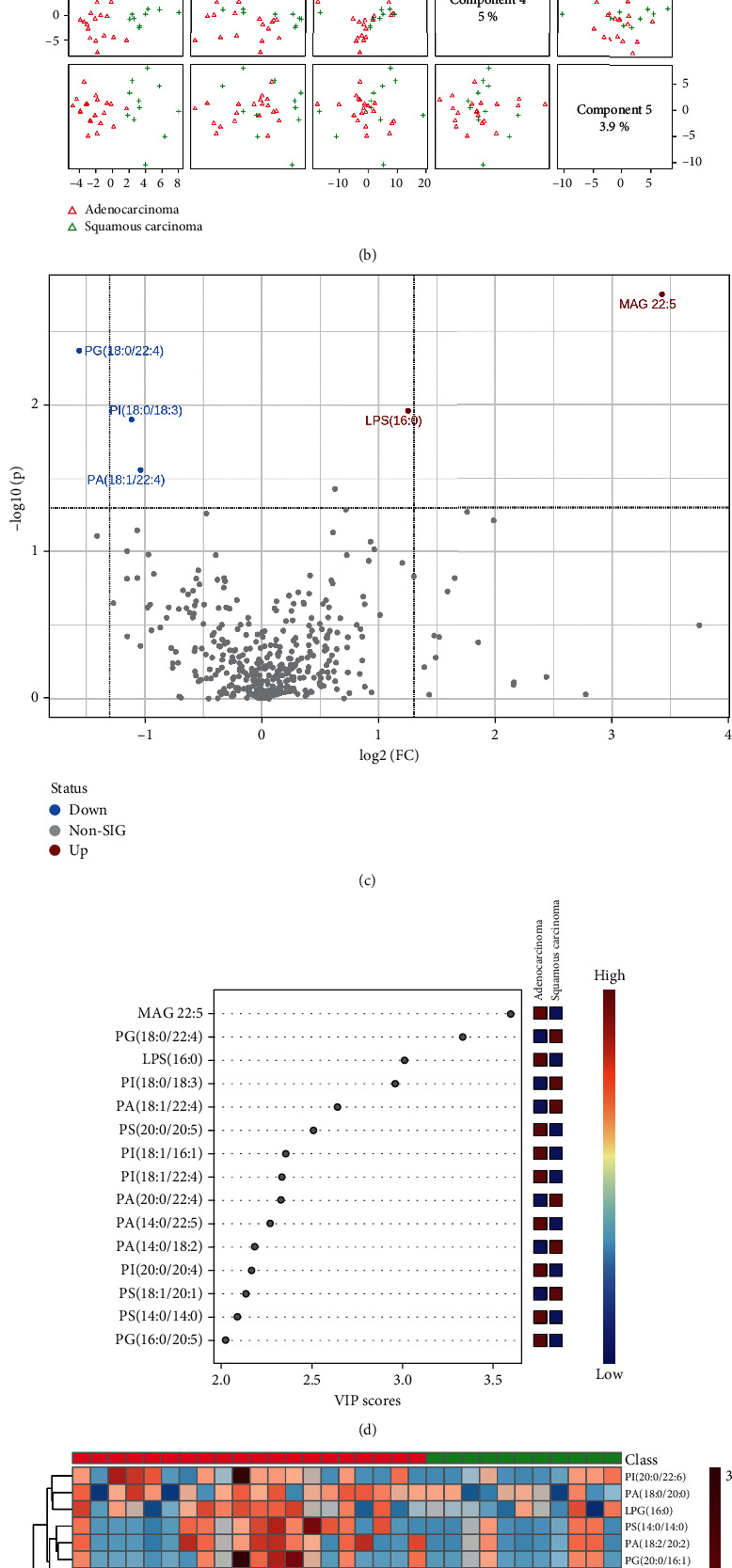
Comparison of alteration of lipid elements between lung adenocarcinoma and lung squamous carcinoma patients during radiotherapy. (a, b) The plots of two-dimensional principal component analysis and five component distributions and percentages show the lipid species and scatters. (c) The volcano shows the changes of lipid elements in the two lung cancer subtypes. (d) The bubble diagram shows the VIP scores of the top 15 lipid elements in the lung adenocarcinoma and lung squamous carcinoma patients. (e) The heat map shows the detailed concentration of lipid elements (top 50).

During radiotherapy, as shown in Figures 6(a) and 6(b), lipid types in NSCLC patients with the two pathological types were also clearly distinguished on two-dimensional principal component analysis maps, and the PLS-DA component analysis exhibited that the five principal components were 4.4%, 12.6%, 15.7%, 5%, and 3.9%.  Figure 6(c) was a visual volcano diagram of lipid elements increased (MAG 22 : 5 and LPG 16 : 0) or decreased (PG (18 : 0/20 : 4), PI (18 : 0/20 : 3), and PA (18 : 1/22 : 4)) by more than two times when the two lung cancer subtypes were compared. As shown in Figure 6(d), the bubble plot via PLS-DA component analysis showed the top 15 lipid elements according to the VIP scores from the comparison between lung adenocarcinoma and lung squamous carcinoma of RT group. We found that there were significant differences in the expression of the top 50 lipid elements between the two subtypes of lung cancer in RT group (Figure 6(e)).

**Figure 7:**
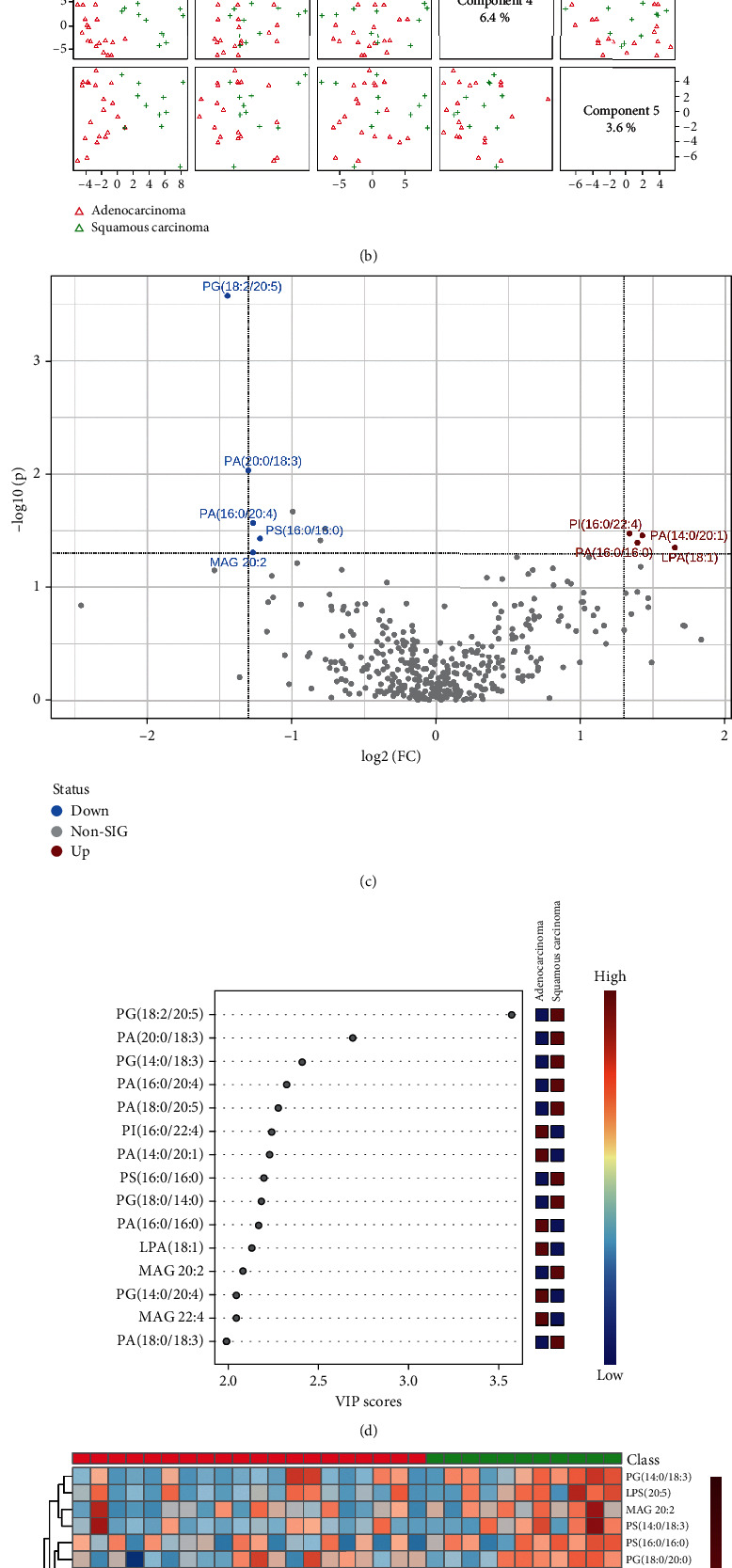
Comparison of alteration of lipid elements between lung adenocarcinoma and lung squamous carcinoma patients after radiotherapy. (a, b) The plots of two-dimensional principal component analysis and five component distributions and percentages show the lipid species and scatters. (c) The volcano shows the changes of lipid elements in the two lung cancer subtypes. (d) The bubble diagram shows the VIP scores of the top 15 lipid elements in the lung adenocarcinoma and lung squamous carcinoma patients. (e) The heat map shows the detailed concentration of lipid elements (top 50).

After radiotherapy, as shown in Figures 7(a) and 7(b), lipid types in NSCLC patients with the two pathological types were also clearly distinguished on two-dimensional principal component analysis maps, and the PLS-DA component analysis exhibited that the five principal components were 7%, 15.1%, 6%, 6.4%, and 3.6%.  Figure 7(c) was a visual volcano diagram of lipid elements elevated or declined by more than two times when the two lung cancer subtypes were compared. As shown in Figure 7(d), the bubble plot via PLS-DA component analysis showed the top 15 lipid elements according to the VIP scores from the comparison between lung adenocarcinoma and lung squamous carcinoma of post-RT group. We found that there were significant differences in the expression of the top 50 lipid elements between the two subtypes of lung cancer in post-RT group (Figure 7(e)).

### 3.4. Different Lipid Omics of NSCLC Patients between Bad Response and Good Response Group

**Figure 8:**
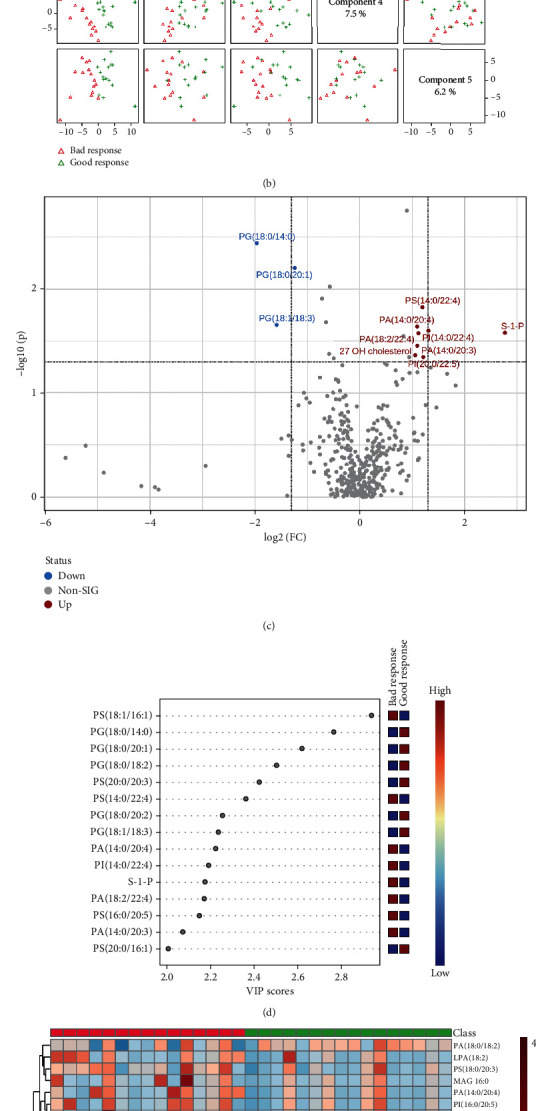
Comparison of alteration of preradiotherapeutic lipid elements of NSCLC patients with bad or good response. (a, b) The plots of two-dimensional principal component analysis and five component distributions and percentages show the lipid species and scatters. (c) The volcano shows the changes of lipid elements in the two different groups. (d) The bubble diagram shows the VIP scores of the top 15 lipid elements in NSCLC patients with bad or good response. (e) The heat map shows the detailed concentration of lipid elements (top 50).

After radiotherapy, we divided the efficacy of patients into CR (complete response), PR (partial response), SD (stable disease), and PD (progressive disease) according to RECIST (Response Evaluation Criteria in Solid Tumors) scoring criteria and defined CR and PR patients as the good response group and SD and PD patients as the bad response group. As compared with good response patients of pre-RT, RT, or post-RT groups, about 7 or 3 lipid elements, about 2 or 2 lipid elements, or about 15 or 0 lipid elements markedly elevated or decreased more than twofold in NSCLC patients with bad response of corresponding group (Table 5). As shown in Figures 8(a) and 8(b), before radiotherapy, lipid types in NSCLC patients with the two response groups were clearly distinguished on two-dimensional principal component analysis maps, and the PLS-DA component analysis exhibited that the five principal components were 10.2%, 12.3%, 5.5%, 7.5%, and 6.2%.  Figure 8(c) was a visual volcano diagram of lipid elements increased or decreased by more than two times when the two response groups were compared. As shown in Figure 8(d), the bubble plot via PLS-DA component analysis showed the top 15 lipid elements according to the VIP scores via the comparison between good response and bad response in NSCLC patients of pre-RT group. We found that as shown in Figure 8(e), the heat map about the concentration of lipid elements showed that there were significant differences in the expression of the top 50 lipid elements between the two therapeutic response types of NSCLC patients in pre-RT group.

**Figure 9:**
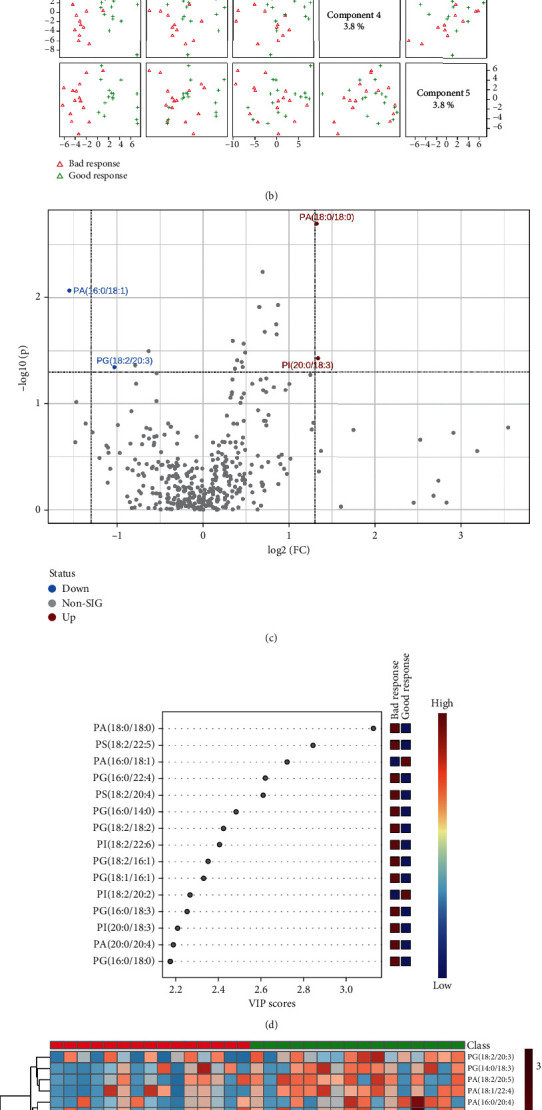
Comparison of alteration of lipid elements of NSCLC patients with bad or good response during radiotherapy. (a, b) The plots of two-dimensional principal component analysis and five component distributions and percentages show the lipid species and scatters. (c) The volcano shows the changes of lipid elements in the two different groups. (d) The bubble diagram shows the VIP scores of the top 15 lipid elements in NSCLC patients with bad or good response. (e) The heat map shows the detailed concentration of lipid elements (top 50).

During radiotherapy, as shown in Figures 9(a) and 9(b), lipid types in NSCLC patients with the two therapeutic response groups were also clearly distinguished on two-dimensional principal component analysis maps, and the PLS-DA component analysis exhibited that the five principal components were 7.3%, 19.2%, 7.1%, 3.8%, and 3.8%.  Figure 9(c) was a visual volcano diagram of lipid elements increased (PA (18 : 0/18 : 0) and PI (20 : 0/18 : 3)) or decreased (PG (18 : 2/20 : 3) and PA (16 : 1/18 : 1)) by more than two times when the NSCLC patients of two response groups were compared. As shown in Figure 9(d), the bubble plot via PLS-DA component analysis showed the top 15 lipid elements according to the VIP scores from the comparison between lung adenocarcinoma and lung squamous carcinoma of RT group. We found that there were significant differences in the expression of the top 50 lipid elements of NSCLC patients between the two response types in RT group (Figure 9(e)).

**Figure 10:**
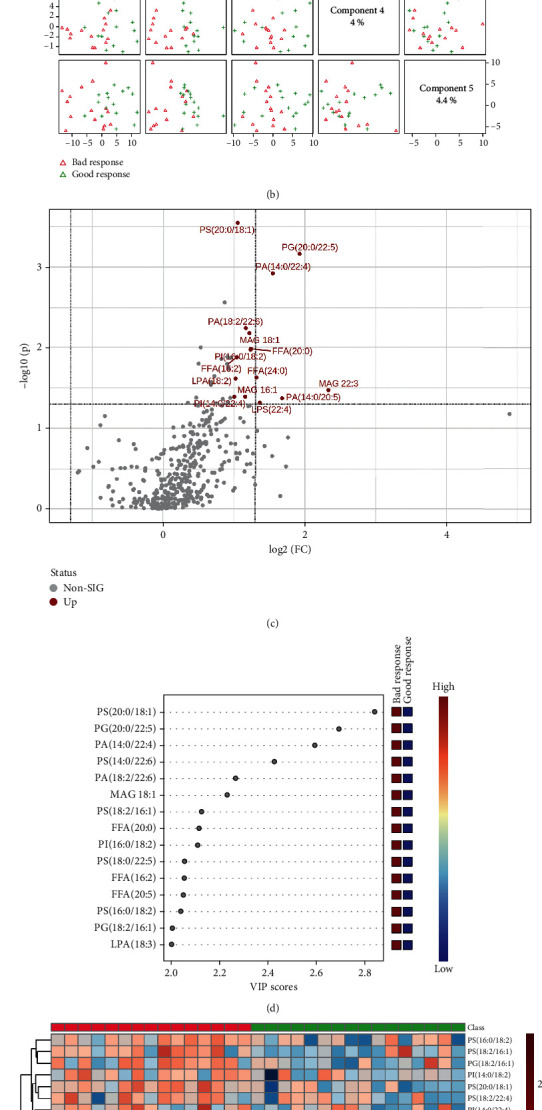
Comparison of alteration of lipid elements of NSCLC patients with bad or good response after radiotherapy. (a, b) The plots of two-dimensional principal component analysis and five component distributions and percentages show the lipid species and scatters. (c) The volcano shows the changes of lipid elements in the two different groups. (d) The bubble diagram shows the VIP scores of the top 15 lipid elements in NSCLC patients with bad or good response. (e) The heat map shows the detailed concentration of lipid elements (top 50).

After radiotherapy, as shown in Figures 10(a) and 10(b), lipid types in NSCLC patients with the two pathological types were also clearly distinguished on two-dimensional principal component analysis maps, and the PLS-DA component analysis exhibited that the five principal components were 17.2%, 6.1%, 7.2%, 4%, and 4.4%.  Figure 10(c) was a visual volcano diagram of lipid elements elevated or declined by more than two times when the NSCLC patients of two response types were compared. As shown in Figure 10(d), the bubble plot via PLS-DA component analysis showed the top 15 lipid elements according to the VIP scores by the comparison of NSCLC patients between good response and bad response after radiotherapy. We found that there were significant differences in the expression of the top 50 of lipid elements of SCLC patients between the two response types after radiotherapy, and the concentrations of the top 50 of lipid elements in NSCLC patients with good response were markedly lower than in NSCLC patients with bad response (Figure 10(e)).

### 3.5. ROC Curve Analysis of Lipid Elements on Radiotherapy Efficacy and Diagnosis of NSCLC Patients

**Figure 11:**
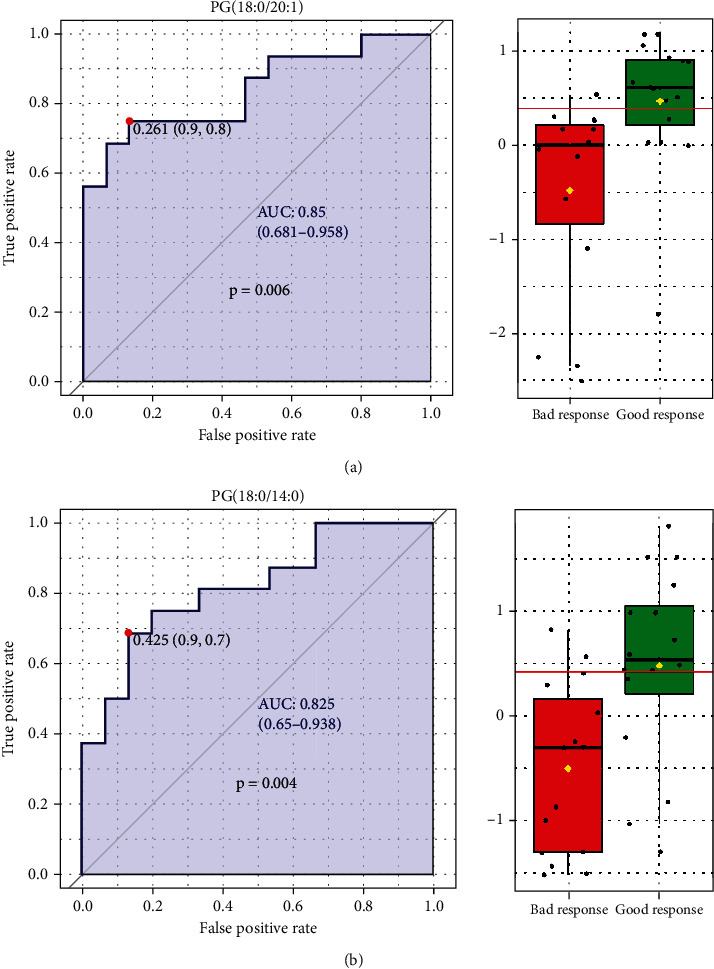
The ROC curve analyses about preradiotherapeutic lipid elements in NSCLC patient's radiotherapy efficacy. (a) ROC curve and box plot of PG (18 : 0/14 : 0). (b) ROC curve and box plot of PG (18 : 0/20 : 0).

**Figure 12:**
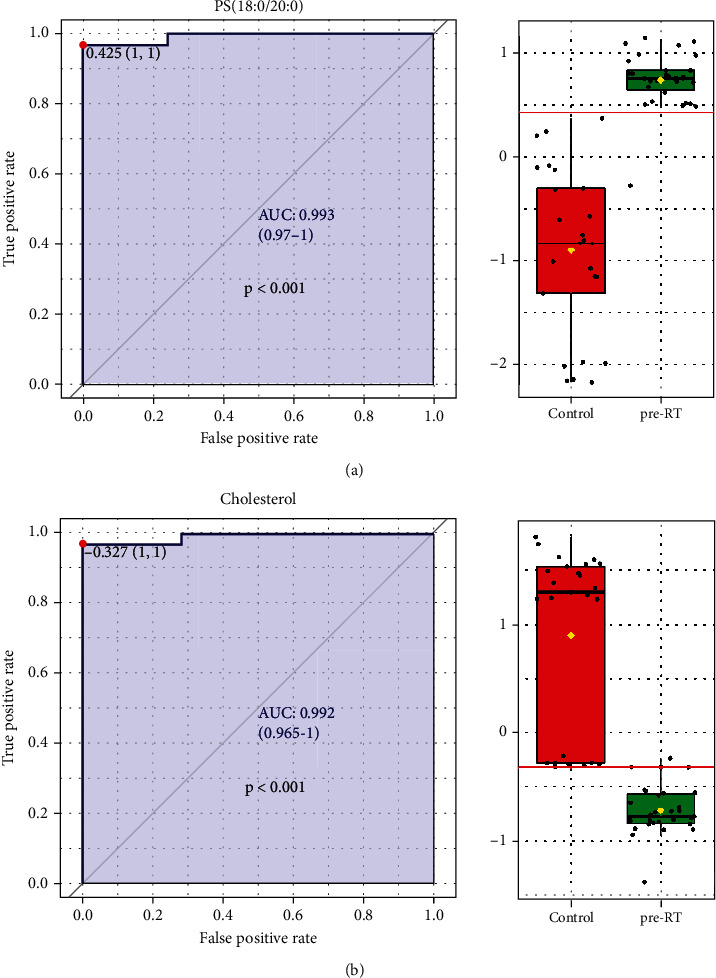
The ROC curve analyses about lipid elements in NSCLC patient's diagnosis. (a) ROC curve and box plot of PS (18 : 0/20 : 0). (b) ROC curve and box plot of cholesterol.

To further investigate the value of lipidomics expression on the predication of radiotherapy efficacy, we used the expression data of preradiotherapeutic lipid elements of NSCLC patients to make a ROC curve analysis, and the results of AUC (area under the curve) and *p* value (*p* < 0.05) of 19 lipid elements were both shown in Table 6. As shown in Figure 11, we found that PG (18 : 0/20 : 1 and 18 : 0/14 : 0) could clearly predict the response of radiotherapy for NSCLC patients, and their AUC were 0.85 (*p* = 0.006) and 0.825 (*p* = 0.004), respectively. In addition, we listed 16 lipid elements of obvious value for lung cancer diagnosis in Table 6, whose AUC was greater than 0.8 and *p* value was less than 0.05. The ROC curve of the first two lipid elements was exhibited in Figure 12, and the results indicated that the AUC of PS (18 : 0/20 : 0) and cholesterol were 0.993 (*p* < 0.001) and 0.992 (*p* < 0.001), respectively, and they were the first two lipid components with the most potential for the diagnosis of NSCLC.

### 3.6. The Enrichment Analysis of Lipid Differential Compounds

**Figure 13:**
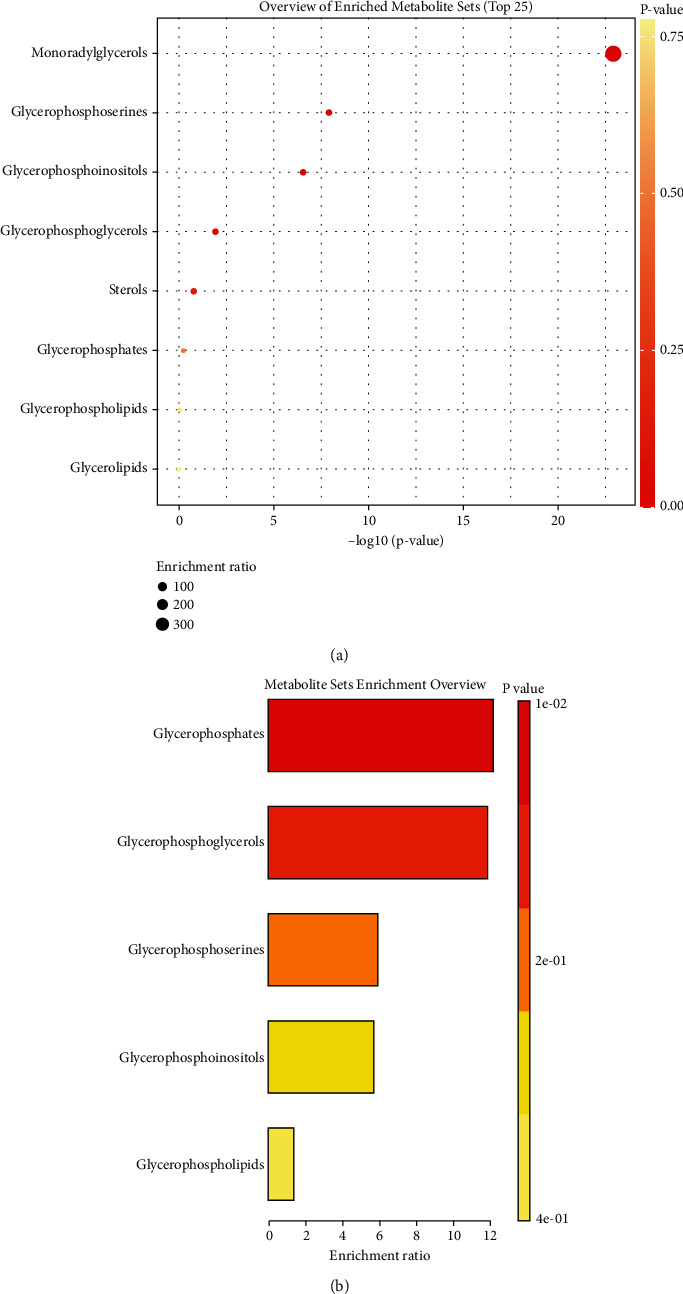
The enrichment analysis of lipid differential compounds. (a) Overview of enriched metabolite sets between NSCLC patients of pre-RT group and healthy controls. (b) Metabolite set enrichment overview between NSCLC patients of pre-RT group with bad and good responses.

In this part, we conducted an enrichment analysis of differential lipid compounds (fold change > 2 and *p* value <0.05) between healthy volunteers and NSCLC patients in pre-RT group (Figure 13(a)), finding that those lipid elements were mainly enriched in monoradylglycerols (enrichment radio = 371.7, *p* < 0.0001). In addition, we also conducted an enrichment analysis of differential lipid compounds (fold change > 2 and *p* value <0.05) between NSCLC patients of pre-RT group with bad and good responses, and the results showed that those lipid elements were mainly enriched in glycerophosphates (enrichment radio = 12.1, *p* = 0.011) and glycerophoglycerols (enrichment radio = 11.8, *p* = 0.011).

## 4. Discussion

In the past decade, lipid metabolomics has been used in various studies to identify tissue, plasma, or serum metabolites to diagnose the occurrence and progression of tumors, including breast cancer [19], prostate cancer [20], kidney cancer [21], and lung cancer [22], which are closely related to metabolic disorders in the body. Recent studies state that plasma lipid species could serve as promising diagnostic markers for early NSCLC patients [23, 24], and serum metabonomics analysis could predict the efficacy of chemotherapy in NSCLC patients [25]. However, there are few studies on the differential value of lipidomics expression in advanced lung cancer. In this study, we found that compared to healthy people, MAG or PI and PS were mainly and obviously declined or elevated lipid species (> twofold, and *p* < 0.05) among advanced NSCLC patients with different radiotherapy phases. The overview of the bubble plot of the top 15 lipid elements showed that compared to other groups, PI (14 : 0/18 : 1), MAG (22 : 0), and PS (20 : 0/20 : 5, 20 : 0/18 : 1, and 20 : 0/22 : 4) decreased in NSCLC patients of pre-RT group; PI (18 : 0/20 : 4) and MAG (20 : 0) decreased in NSCLC patients of RT group; PS (18 : 0/20 : 0 and 20 : 0/16 : 1), cholesterol, MAG (22 : 5, 20 : 2, and 22 : 3), and PI (16 : 0/18 : 3 and 18 : 1/18 : 2) decreased in NSCLC patients of post-RT group. Moreover, the heat map data showed that the concentrations of lipid types in healthy volunteers were significantly different from those in advanced NSCLC patients at different stages of radiotherapy. These differences in lipid elements between advanced NSCLC patients and healthy individuals would help to screen for specific diagnostic markers. Further investigation in our research found that 16 lipid elements of obvious value (AUC > 0.8, *p* < 0.05) could be used for NSCLC diagnosis in Table 6, and Figure 12 lists the first two lipid components with the most potential for the diagnosis of NSCLC and showed the best cutoff value, respectively. Therefore, it was shown in the present study that plasma lipidomics were used to identify the markers of advanced NSCLC patients, leading to early active and effective intervention to improve quality of life and prolong survival.

As we all know, lung cancer consisted in small cell lung cancer (SCLC) and non-small-cell lung cancer (NSCLC) according to biological behaviors, whereas NSCLC were mainly divided into lung adenocarcinoma (AC) and lung squamous carcinoma (SC) according to pathological typing. Many researches indicated that different types of lung cancer may have different metabolic disorders and have different biomarkers for specific diagnosis [26, 27], which was of great significance for the specific and precise treatment of lung cancer. Researchers used high-resolution magic angle spinning (HRMAS) NMR spectroscopy to analyze the matched tumor and adjacent control tissues from 56 patients undergoing surgical excision of primary lung carcinomas, finding that major alterations in AC were related to phospholipid metabolism, whereas main changes were associated to glycolytic and glutaminolytic profiles in SC [28]. Additionally, the results of untargeted metabolomics of other researchers showed that glycerophospho (N-acyl) ethanolamines could discriminate early-stage AC and SC in NSCLC patients' tissue [22]. Further investigation in detail showed that levels of PE elements (36 : 2, 18 : 0/18 : 2, and 18 : 1/18 : 1) were found to be specific for SCLC, and lysoPC (20 : 1 and 22 : 0 sn-position-1) and PC (19 : 0/19 : 0 and PC 19 : 0/21 : 2) were specific for AC [16]. In our study, we also found a significant difference in lipid expression profile between advanced lung adenocarcinoma and lung squamous cell carcinoma, which was consistent with the results of previous studies on early lung adenocarcinoma and lung squamous cell carcinoma, indicating that whether early lung cancer or advanced lung cancer, lipid expression profile is of great value for differentiating the pathological types of lung cancer. At the same time, it would also provide new insights and ideas for the specific treatment of advanced lung cancer.

In recent years, studies have shown that metabolomics can identify radiation-induced molecular targets and related signaling pathways, thereby providing more information about cellular physiological changes, which has great potential in biomarker screening [29]. Furthermore, metabolomics studies have partially validated the classical pathways of radiation damage, including oxidative stress and subsequent DNA breakdown, and the metabolite panel included LPC (20 : 3), LPC (20 : 2), PC (18 : 0/22 : 5), L-palmitoylcarnitine, N-acetylornithine, and butyrylcarnitine that may be potential biomarkers for the rapid classification of radiation injury [30]. Our studies exhibited that as compared with good response patients of pre-RT, RT, or post-RT groups, about 10 lipid elements, about 4 lipid elements, or about 15 lipid elements markedly altered more than twofold in NSCLC patients with bad response of corresponding group, and as can be seen from the scatter plots and figures of principal component analysis, lipid expression profiles of lung cancer patients in the groups with good and bad radiotherapy response were significantly different. These differences in lipid expression at different stages of radiotherapy will contribute to further screening of potential markers for predicting radiotherapy response in advanced lung cancer. In our in-depth research, the results of ROC curve analysis indicated that PG (18 : 0/20 : 1 and 18 : 0/14 : 0), as the first two lipid components, could clearly predict the response of radiotherapy for NSCLC patients, and their AUC were 0.85 (*p* = 0.006) and 0.825 (*p* = 0.004), respectively. Moreover, our enrichment analysis of those potential lipid elements showed that they were mainly enriched in glycerophosphates (enrichment radio = 12.1, *p* = 0.011) and glycerophoglycerols (enrichment radio = 11.8, *p* = 0.011), which suggested that glycerophospholipid metabolism may be associated with the response to radiotherapy in advanced NSCLC patients, and this finding was consistent with previous metabolomics studies of radiation damage [30].

Glycerophospholipid was the most abundant phospholipid in living organisms and was an important component of biofilm and bile, which played an important role in protein recognition and signal transduction. Therefore, combined with the results of our study, it could be found that targeting the pathway of phospholipid glycerol metabolism would provide a new idea and direction for increasing the efficacy of radiotherapy in NSCLC patients. However, there are some limitations to our study. On the one hand, the sample size is small, and it is a single-center study. We need to further expand the sample size and carry out multicenter studies to verify our results. On the other hand, more animal models and cellular and molecular biology experiments are needed to verify the predictive role of lipid metabolomics expression profile in the diagnosis and the efficacy of radiotherapy of advanced lung cancer.

## 5. Conclusion

In summary, we characterized the altered and varied lipid elements and concentrations among NSCLC patients with different radiotherapy phases, NSCLC subtypes, and different radiotherapeutic responses. Our results concluded that plasma lipidomics profiling might have a vital value to uncover the heterogeneity of lipid metabolism in healthy people and advanced NSCLC patients with different radiotherapy phase and further to screen out the specific biomarkers of advanced NSCLC diagnosis and radiotherapeutic response.

## Figures and Tables

**Table 1:** More than twofold declined lipid elements of NSCLC patients in pre-RT, RT, and post-RT groups with statistical significance, as compared with healthy control (*p* < 0.05 or less).

Pre-RT/Con			RT/Con			Post-RT/Con		
Elements	Fold change	*p* value	Elements	Fold change	*p* value	Elements	Fold change	*p* value
MAG 22 : 3	0.001	<0.001	MAG 20 : 2	0.001	<0.001	MAG 22 : 0	0.002	<0.001
MAG 22 : 0	0.002	<0.001	MAG 22 : 0	0.002	<0.001	MAG 22 : 3	0.002	<0.001
MAG 20 : 0	0.002	<0.001	27 OH cholesterol	0.002	0.006	27 OH cholesterol	0.002	0.007
MAG 20 : 2	0.002	<0.001	MAG 20 : 4	0.002	<0.001	MAG 22 : 5	0.002	<0.001
MAG 20 : 1	0.003	<0.001	MAG 22 : 6	0.003	0.006	MAG 20 : 4	0.002	<0.001
27 OH cholesterol	0.003	0.016	Cholesterol	0.003	<0.001	MAG 22 : 6	0.002	0.008
MAG 22 : 6	0.003	0.008	MAG 22 : 4	0.004	0.009	MAG 20 : 0	0.003	<0.001
MAG 22 : 1	0.003	<0.001	MAG 18 : 2	0.005	0.002	MAG 20 : 2	0.003	<0.001
Cholesterol	0.003	<0.001	MAG 22 : 5	0.007	<0.001	MAG 20 : 3	0.003	0.004
MAG 18 : 0	0.006	0.009	MAG 20 : 1	0.008	<0.001	MAG 20 : 1	0.003	<0.001
MAG 18 : 1	0.007	0.001	MAG 22 : 1	0.008	0.002	MAG 22 : 4	0.003	0.002
MAG 22 : 5	0.021	<0.001	MAG 20 : 0	0.010	<0.001	Cholesterol	0.003	<0.001
MAG 20 : 4	0.022	<0.001	MAG 22 : 3	0.010	<0.001	MAG 22 : 1	0.003	<0.001
MAG 22 : 2	0.023	0.001	MAG 22 : 2	0.015	0.001	MAG 22 : 2	0.003	<0.001
MAG 22 : 4	0.039	0.004	MAG 18 : 1	0.015	0.008	MAG 18 : 2	0.004	0.001
MAG 18 : 2	0.061	0.006	MAG 18 : 0	0.016	0.005	MAG 18 : 0	0.005	0.001
PS (18 : 1/22 : 4)	0.298	<0.001	FFA(24 : 0)	0.312	<0.001	MAG 18 : 1	0.006	0.001
FFA (24 : 0)	0.336	<0.001	PS (18 : 1/22 : 4)	0.313	<0.001	PS (18 : 1/22 : 4)	0.215	<0.001
PI (18 : 1/20 : 2)	0.410	0.029	PS (14 : 0/20 : 5)	0.347	0.007	FFA (24 : 0)	0.251	<0.001
PG (20 : 0/18 : 3)	0.417	0.001	PI (18 : 2/22 : 4)	0.386	0.003	PI (18 : 1/20 : 2)	0.305	0.022
PI (20 : 0/20 : 5)	0.424	0.032	PG (18 : 1/18 : 3)	0.438	0.001	PI (18 : 0/20 : 1)	0.350	0.007
PG (18 : 1/18 : 3)	0.429	0.001	PG (20 : 0/20 : 3)	0.458	0.012	LPG (20 : 0)	0.393	0.047
PI (18 : 2/22 : 4)	0.433	0.001	PS (16 : 0/22 : 6)	0.471	0.002	PI (18 : 2/22 : 4)	0.418	0.002
PS (18 : 0/22 : 6)	0.437	0.002	PG (20 : 0/20 : 2)	0.475	0.028	PG (18 : 0/22 : 6)	0.430	0.001
PG (20 : 0/20 : 1)	0.461	0.022				PI (20 : 0/22 : 6)	0.445	0.030
PI (20 : 0/22 : 6)	0.466	0.028				PG (20 : 0/20 : 1)	0.469	0.033
PI (16 : 0/16 : 0)	0.471	0.006				PS (14 : 0/20 : 5)	0.498	0.032
PA (14 : 0/18 : 3)	0.480	0.012						
PI (18 : 2/20 : 5)	0.484	0.003						
PS (14 : 0/20 : 3)	0.494	0.028						

NSCLC: non-small-cell lung cancer; pre-RT: preradiotherapy; RT: radiotherapy; post-RT: postradiotherapy.

**Table 2:** More than twofold elevated lipid elements of NSCLC patients in pre-RT, RT, and post-RT groups with statistical significance, as compared with healthy control (*p* < 0.05 or less).

Pre-RT/Con			RT/Con			Post-RT/Con		
Elements	Fold change	*p* value	Elements	Fold change	*p* value	Elements	Fold change	*p* value
PS (20 : 0/20 : 1)	2.200	0.015	PA (20 : 0/16 : 1)	2.090	0.016	PG (18 : 0/16 : 1)	2.243	0.034
PI (20 : 0/22 : 5)	2.269	0.022	PI (18 : 1/18 : 1)	2.120	0.005	PS (18 : 0/16 : 1)	2.335	<0.001
PS (18 : 0/16 : 1)	2.391	<0.001	PA (18 : 0/20 : 2)	2.223	0.003	PI (18 : 0/22 : 4)	2.361	0.022
PS (20 : 0/18 : 1)	2.395	<0.001	PI (16 : 0/18 : 1)	2.394	0.001	PI (18 : 1/18 : 2)	2.371	0.012
LPS (16 : 0)	2.494	0.003	PI (18 : 0/18 : 1)	2.490	<0.001	PS (20 : 0/20 : 1)	2.453	0.006
LPS (18 : 0)	2.511	0.003	PI (18 : 0/16 : 1)	2.621	0.003	PI (18 : 0/18 : 1)	2.561	0.041
PS (20 : 0/16 : 1)	2.541	<0.001	PG (18 : 1/18 : 2)	2.640	0.001	PS (20 : 0/18 : 1)	2.565	<0.001
PG (16 : 0/16 : 1)	2.686	0.032	PS (20 : 0/18 : 1)	2.644	<0.001	PS (20 : 0/16 : 1)	2.592	<0.001
PG (18 : 0/16 : 1)	2.785	0.001	PG (16 : 0/16 : 1)	2.679	<0.001	PS (18 : 0/14 : 0)	2.597	0.013
PS (14 : 0/22 : 5)	3.144	0.002	PS (20 : 0/16 : 1)	2.818	<0.001	PG (16 : 0/16 : 1)	2.686	0.002
PI (18 : 0/20 : 3)	3.316	0.001	PI (18 : 0/20 : 2)	2.855	0.019	PG (18 : 1/18 : 2)	2.778	<0.001
PS (18 : 0/14 : 0)	3.821	0.001	LPS (18 : 0)	2.968	<0.001	PI (18 : 0/20 : 3)	2.864	0.006
PS (20 : 0/20 : 5)	5.192	<0.001	PI (18 : 1/18 : 2)	3.056	<0.001	LPS (18 : 0)	2.897	0.001
PI (14 : 0/18 : 1)	6.047	<0.001	PG (18 : 0/16 : 1)	3.088	<0.001	PI (16 : 0/18 : 1)	2.937	0.003
PS (18 : 0/20 : 0)	15.236	<0.001	PA (16 : 0/18 : 1)	3.629	0.004	PG (18 : 1/18 : 1)	2.994	0.001
PI (18 : 0/20 : 4)	30.836	<0.001	PI (18 : 0/20 : 3)	3.890	0.001	PA (16 : 0/18 : 1)	4.468	0.001
			PS (20 : 0/20 : 5)	5.264	<0.001	PS (20 : 0/20 : 5)	5.034	<0.001
			PI (14 : 0/18 : 1)	8.042	<0.001	PI (14 : 0/18 : 1)	6.531	<0.001
			PS (18 : 0/20 : 0)	14.602	<0.001	PS (18 : 0/20 : 0)	13.548	<0.001
			PI(18 : 0/20 : 4)	21.894	<0.001	PI(18 : 0/20 : 4)	24.485	<0.001

NSCLC: non-small-cell lung cancer; pre-RT: preradiotherapy; RT: radiotherapy; post-RT: postradiotherapy.

**Table 3:** More than twofold declined or elevated lipid elements of NSCLC patients in pre-RT, RT, or post-RT groups with statistical significance, as compared with each other (*p* < 0.05 or less).

RT/pre-RT			Post-RT/pre-RT			Post-RT/RT		
Elements	Fold change	*p* value	Elements	Fold change	*p* value	Elements	Fold change	*p* value
PS (14 : 0/20 : 5)	0.433	0.010	PS (14 : 0/22 : 5)	0.425	0.001	PA (18 : 2/22 : 5)	0.386	0.025
PS (14 : 0/22 : 5)	0.488	0.020	PG (18 : 2/20 : 5)	0.481	0.020	MAG 20 : 1	0.387	0.032
PA (14 : 0/20 : 2)	2.243	0.038	PI (18 : 2/20 : 1)	2.038	0.007	PA (14 : 0/20 : 2)	0.426	0.025
PA (18 : 1/20 : 2)	2.254	0.050	PS (14 : 0/20 : 3)	2.230	0.029	LPS (18 : 1)	0.443	0.019
PA (14 : 0/18 : 3)	2.832	0.020	PA (16 : 0/18 : 1)	2.281	0.021	LPG (20 : 0)	0.470	0.003
MAG 22 : 3	8.515	0.050				PA (18 : 2/18 : 2)	0.472	0.021
						PI (18 : 0/20 : 1)	0.474	0.044
						PG (18 : 1/22 : 6)	2.017	0.024

NSCLC: non-small-cell lung cancer; pre-RT: preradiotherapy; RT: radiotherapy; post-RT: postradiotherapy.

**Table 4:** More than twofold declined or elevated lipid elements of NSCLC patients with adenocarcinoma and squamous carcinoma in pre-RT, RT, or post-RT groups (*p* < 0.05 or less).

AC/SC pre-RT			AC/SC RT			AC/SC post-RT		
Elements	Fold change	*p* value	Elements	Fold change	*p* value	Elements	Fold change	*p* value
PA (16 : 0/22 : 5)	0.334	0.006	PG (18 : 0/22 : 4)	0.339	0.004	PG (18 : 2/20 : 5)	0.367	<0.001
FFA (20 : 5)	0.356	0.034	PI (18 : 0/18 : 3)	0.462	0.013	PA (20 : 0/18 : 3)	0.405	0.009
PA (18 : 2/20 : 2)	0.399	0.002	PA (18 : 1/22 : 4)	0.488	0.028	MAG 20 : 2	0.415	0.049
PG (20 : 0/22 : 4)	0.444	0.018	LPS (16 : 0)	2.383	0.011	PA (16 : 0/20 : 4)	0.415	0.027
PG (20 : 0/20 : 2)	0.451	0.013	MAG 22 : 5	10.759	0.002	PS (16 : 0/16 : 0)	0.429	0.037
PA (18 : 1/22 : 4)	0.457	0.008				PI (16 : 0/22 : 4)	2.528	0.033
PS (20 : 0/20 : 5)	2.416	0.006				PA (16 : 0/16 : 0)	2.625	0.040
PS (18 : 2/20 : 5)	2.845	0.019				PA (14 : 0/20 : 1)	2.687	0.035
						LPA (18 : 1)	3.144	0.044

AC: adenocarcinoma; SC: squamous carcinoma; NSCLC: non-small-cell lung cancer; pre-RT: preradiotherapy; RT: radiotherapy; post-RT: postradiotherapy.

**Table 5:** More than twofold declined or elevated lipid elements of NSCLC patients with bad response and good response in pre-RT, RT, or post-RT groups (*p* < 0.05 or less).

Bad/good response pre-RT			Bad/good response RT			Bad/good response post-RT		
Elements	Fold change	*p* value	Elements	Fold change	*p* value	Elements	Fold change	*p* value
PG (18 : 0/14 : 0)	0.256	0.004	PA (16 : 0/18 : 1)	0.341	0.009	PI (14 : 0/22 : 4)	2.003	0.041
PG (18 : 1/18 : 3)	0.333	0.022	PG (18 : 2/20 : 3)	0.490	0.045	LPA (18 : 2)	2.029	0.024
PG (18 : 0/20 : 1)	0.423	0.006	PA (18 : 0/18 : 0)	2.496	0.002	FFA (16 : 2)	2.053	0.013
27 OH cholesterol	2.084	0.043	PI (20 : 0/18 : 3)	2.525	0.037	PS (20 : 0/18 : 1)	2.072	<0.001
PA (14 : 0/20 : 4)	2.138	0.023				MAG 16 : 1	2.229	0.041
PA (14 : 0/20 : 3)	2.141	0.035				PA (18 : 2/22 : 6)	2.243	0.006
PA (18 : 2/22 : 4)	2.174	0.027				MAG 18 : 1	2.323	0.007
PS (14 : 0/22 : 4)	2.290	0.015				PI (16 : 0/18 : 2)	2.351	0.011
PI (20 : 0/22 : 5)	2.319	0.045				FFA (20 : 0)	2.355	0.010
PI (14 : 0/22 : 4)	2.480	0.025				FFA (24 : 0)	2.490	0.024
						LPS (22 : 4)	2.572	0.048
						PA (14 : 0/22 : 4)	2.921	0.001
						PA (14 : 0/20 : 5)	3.199	0.043
						PG (20 : 0/22 : 5)	3.799	0.001
						MAG 22 : 3	5.027	0.034

NSCLC: non-small-cell lung cancer; pre-RT: preradiotherapy; RT: radiotherapy; post-RT: postradiotherapy.

**Table 6:** ROC curve analysis of NSCLC patients and healthy controls.

Bad/good response pre-RT			NSCLC pre-RT/Con		
Elements	AUC	*p* value	Elements	AUC	*p* value
PG (18 : 0/20 : 1)	0.850	0.006	PS (18 : 0/20 : 0)	0.993	<0.001
PG (18 : 0/14 : 0)	0.825	0.004	Cholesterol	0.992	<0.001
PS (18 : 1/16 : 1)	0.796	0.002	PI (18 : 0/20 : 4)	0.969	<0.001
PG (18 : 1/18 : 3)	0.775	0.022	PI (14 : 0/18 : 1)	0.957	<0.001
PG (18 : 0/18 : 2)	0.767	0.009	MAG 22 : 5	0.956	<0.001
PS (16 : 0/18 : 2)	0.750	0.045	MAG 22 : 0	0.914	<0.001
PS (14 : 0/22 : 4)	0.746	0.015	PS (20 : 0/16 : 1)	0.874	<0.001
PS (20 : 0/20 : 3)	0.729	0.012	MAG 22 : 3	0.863	<0.001
PG (18 : 0/20 : 2)	0.729	0.021	MAG 20 : 0	0.845	<0.001
S-1-P	0.729	0.026	FFA(24 : 0)	0.839	<0.001
PI (14 : 0/22 : 4)	0.725	0.025	PS (20 : 0/20 : 5)	0.833	<0.001
27 OH cholesterol	0.725	0.043	PS (18 : 1/22 : 4)	0.830	<0.001
PA (14 : 0/20 : 4)	0.721	0.023	PS (18 : 0/18 : 0)	0.818	<0.001
PA (18 : 2/22 : 4)	0.717	0.027	PG (16 : 0/18 : 1)	0.810	<0.001
PS (16 : 0/20 : 5)	0.713	0.028	PS (18 : 0/16 : 1)	0.809	<0.001
PA (18 : 0/20 : 5)	0.713	0.046	PS (18 : 0/20 : 3)	0.806	<0.001
PS (20 : 0/16 : 1)	0.700	0.042			
PI (20 : 0/22 : 5)	0.692	0.045			
PA (14 : 0/20 : 3)	0.688	0.035			

NSCLC: non-small-cell lung cancer; pre-RT: preradiotherapy; AUC: area under the curve.

## Data Availability

The data in this study is available from the corresponding author upon reasonable request.

## Supplementary Materials

Supplementary Materials The original data for all analyses in this manuscript are provided in the supplementary document, according to the corresponding subgroups. The corresponding subgroups can be obtained according to the supplementary file name.
